# Esophageal perforation following pedicle screw placement for the treatment of upper thoracic spinal tuberculosis: a case report and review of the literature

**DOI:** 10.1186/s12891-020-03783-4

**Published:** 2020-11-18

**Authors:** Yuhang Wang, Dingjun Hao, Lixiong Qian, Xin He, Yibin Meng, Biao Wang

**Affiliations:** 1 Department of Spine Surgery, Honghui Hospital, Xi’an Jiaotong University College of Medicine, No. 76 Nanguo Road, Xi’an, 710054 Shaanxi China; 2 Xi’an Medical University, No. 74 Hanguang North Road, Xi’an, 710054 Shaanxi China

**Keywords:** Upper thoracic spinal tuberculosis, Esophageal perforation, Esophageal fistula, Pedicle screw, Infection

## Abstract

**Background:**

The technique of posterior pedicle screw fixation has already been widely applied in the treatment of upper thoracic spinal tuberculosis. However, lesions of tuberculosis directly invade the vertebrae and surrounding soft tissues, which increases the risk of esophageal perforation induced by the posterior pedicle screw placement. Herein, we report the first case of esophageal perforation following pedicle screw placement in the upper thoracic spinal tuberculosis, and describe the underlying causes, as well as the treatment and prognosis.

**Case presentation:**

A 48-year-old female patient with upper thoracic spinal tuberculosis presented sputum-like secretions from the wound after she was treated with one-stage operation through the posterolateral approach. Endoscopy was immediately conducted, which confirmed that the patient complicated with postoperative esophageal perforation caused by screws. CT scan showed that the right screw perforated the anterior cortex of the vertebrae and the esophagus at the T4 level. Fortunately, mediastinal infection was not observed. The T4 screw was removed, Vacuum Sealing Drainage (VSD) was performed, and jejunum catheterization was used for enteral nutrition. After continuous treatment with sensitive antibiotics for 2.5 months and 5 times of VSD aspiration, the infected wound recovered gradually. With 18-month follow-up, the esophagus healed well, without symptoms of dysphagia and stomach discomfort, and CT scan showed that T2–4 had complete osseous fusion without sequestrum.

**Conclusion:**

Tuberculosis increases the risk of postoperative esophageal perforation in a certain degree for patients with upper thoracic tuberculosis. The damages to esophagus during the operation should be prevented. The screws with the length no more than 30 mm should be selected. Moreover, close monitoring after operation should be conducted to help the early identification, diagnosis and treatment, which could help preventing the adverse effects induced by the delayed diagnosis and treatment of esophageal perforation.

## Background

Spinal tuberculosis is the most common form of extrapulmonary tuberculosis, accounting for approximately 1 to 3% of all tuberculosis cases, and 50% of musculoskeletal infections [1, 2]. Although relatively rare in clinical practices, upper thoracic tuberculosis could induce severe damages. Patients with upper thoracic spinal tuberculosis are generally accompanied with the destruction of vertebral body and intervertebral disc, formation of paravertebral abscess, and neurological impairments [3]. Among the surgical treatments of upper thoracic tuberculosis, the posterolateral approach with pedicle screw fixation could achieve better correction of kyphosis and reconstruction of spinal stability, and thus has been more and more applied in recent years [4]. However, the complications of nerves, blood vessels, and vital organs caused by the pedicle screws have also attracted increasing attentions [5–7].

Soft tissues such as aorta, esophagus and lung are adjacent to thoracic vertebrae, and the narrowness and inconsistent shapes of the pedicles could also increase the challenges of posterior pedicle screw placement in upper thoracic spine [8, 9]. In addition, lesions of tuberculosis directly invade the vertebral body and surrounding soft tissues, which further increases the risks of vascular, spinal cord, and esophageal complications induced by posterior pedicle screw placement in upper thoracic spine.

Various studies have already reported the neurological and vascular complications related to pedicle screw placement in upper thoracic spine [6, 10–12]. However, the related complications in esophagus, an important structure close to upper thoracic spine, are very rare, and thus only very few cases have been reported. In this case report, we described esophageal perforation caused by pedicle screw in a patient of upper thoracic spinal tuberculosis during the treatment.

## Case presentation

**Fig. 1:**
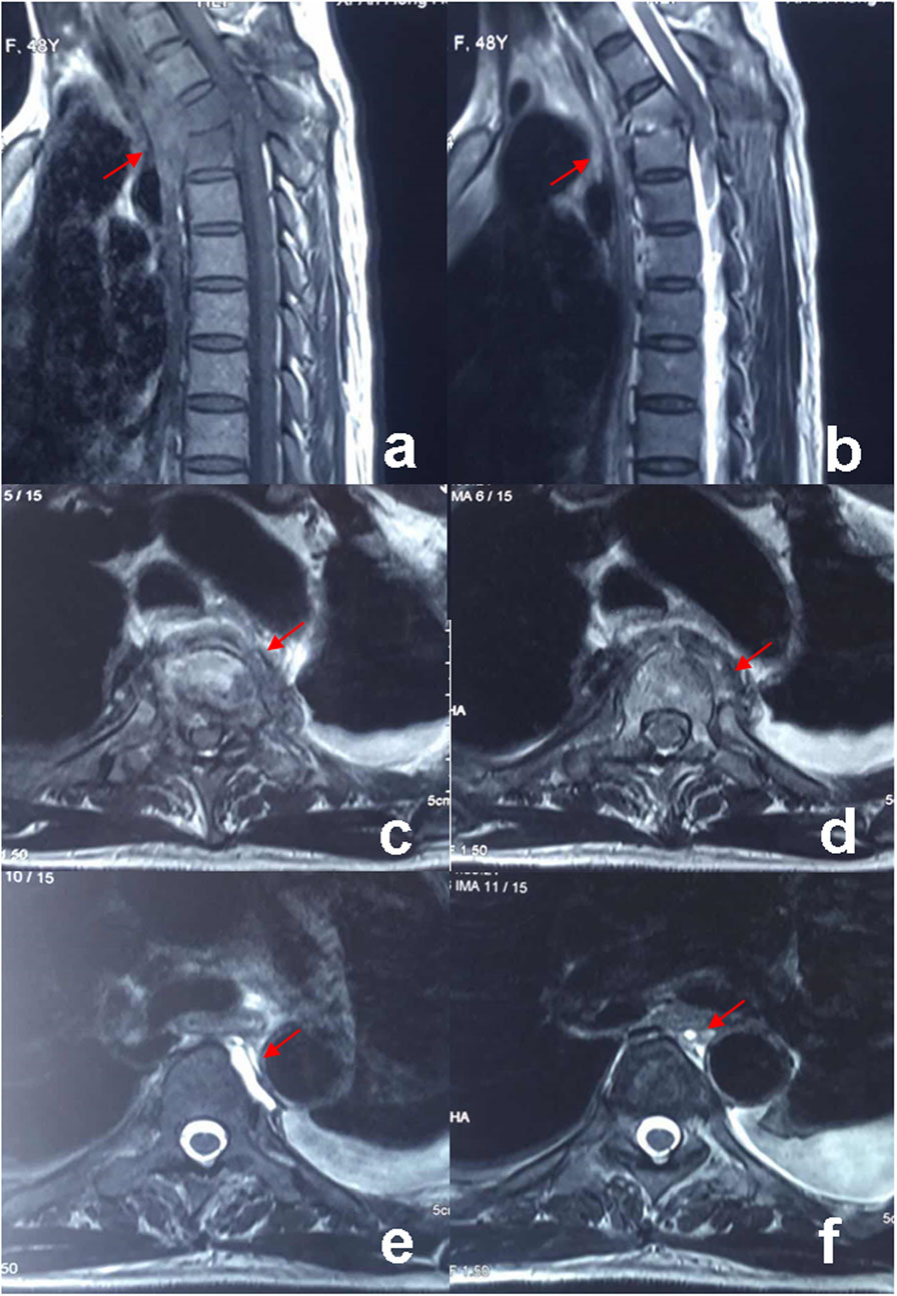
T1 (**a**) and T2 (**b**) sequences of MRI showed T3 / 4 vertebral infectious disease with spinal cord compression, and axial images (**c**-**f**) showed bone defects in the anterior part of T3 and T4 vertebrae, tuberculosis foci were located around the oesophagus and pushed the oesophagus to the right side (the red arrow indicates the location of the lesion)

A 48-year-old female patient was admitted to the hospital due to back pain with unknown causes for over 20 days, which could not be alleviated by nonsteroidal anti-inflammatory drugs (NSAIDs). MRI scan showed abnormal signals at the third and fourth thoracic vertebral bodies and the T3/4 intervertebral disc, as well as infectious lesions and formation of paravertebral abscess (Fig. 1). Blood routine examination of the patient showed no abnormality, of which the erythrocyte sedimentation rate (ESR) was 98 mm/h, C-reactive protein (CRP) was 75 mg/L, T-STOP.TB test showed high positivity. According to international standard revised by American Spinal Cord Injury Association (ASIA), the patient’s neurological function was grade D. Therefore, the patient was diagnosed upper thoracic spinal tuberculosis accompanied with spinal cord injury. The patient was then treated with enhanced tetrad anti-tuberculosis strategy (including isoniazid, rifampicin, pyrazinamide, and streptomycin) for 2 weeks, after which the ESR reduced to 42 mm/h and CRP reduced to 17 mg/L. Then the one-stage operation through the posterolateral approach was conducted. In brief, the focus debridement through intertransverse articulation at the left rib, spinal decompression, and ilium grafting were conducted. The two ends of the lesion were fixed by two groups of pedicle screws to restore the stability of upper thoracic spine and correct the kyphosis. Few tissues of the lesions were obtained during the operation for pathological examinations, which showed caseous necrosis and tuberculosis infection.

After surgery, the patient was in persistent low-grade fever, and the white blood cell count, ESR, and CRP were slightly increased. Such changes were initially considered to be the transient manifestations after operations of infectious diseases. However, sputum-like secretions were found during the dressing on the postoperative Day 7, and the secretions containing small bubbles were found when pressing the wound. Based on our experience of treating esophageal injuries after anterior cervical surgery, the tracheal or esophageal damages were highly suspected, due to the fact that the bubbles could not be found in regular wound infections (except for the infection of aerobacter aerogenes).

**Fig. 2:**
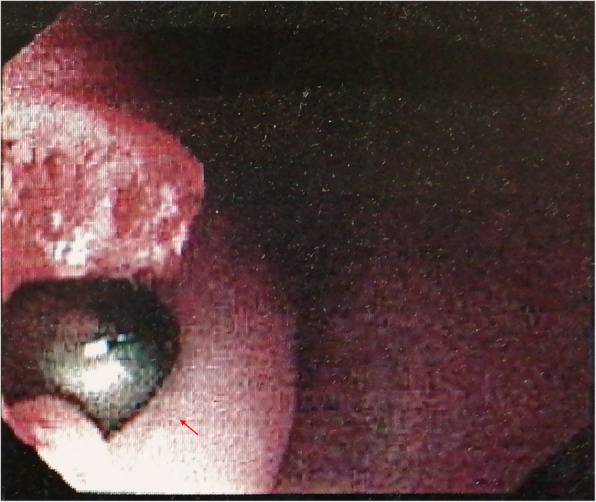
Endoscopy showed oesophageal perforation caused by pedicle screw

**Fig. 3:**
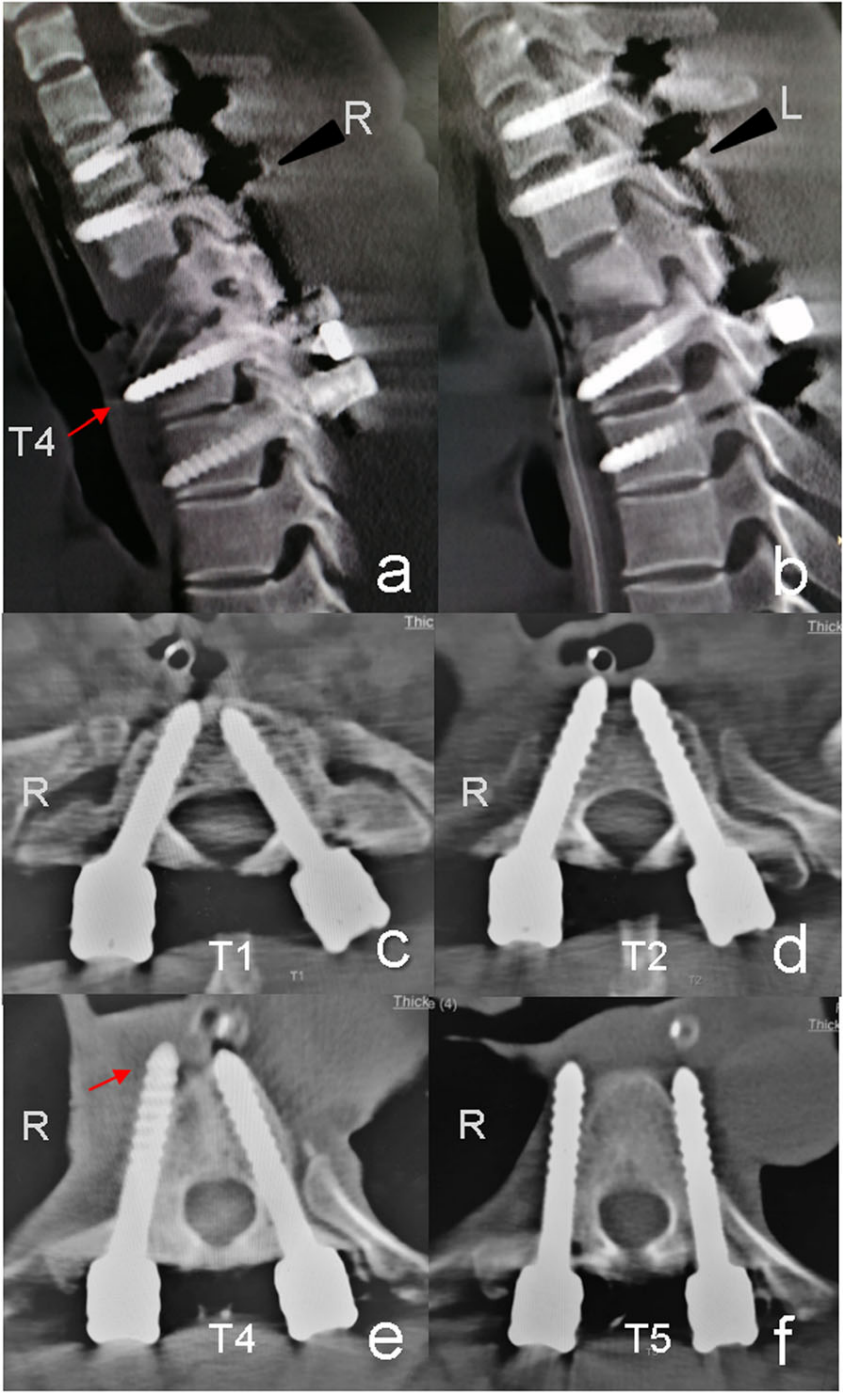
CT examination showed that the right screw of T4 vertebra was too long, penetrated the anterior edge of vertebra and occupied the anatomical position of oesophagus (red arrow), sagittal cuts along with direction of the screws were showed in **a**-**b** and axial cuts at all levels were showed in **c**-**f**

Fiberoptic bronchoscopy was immediately conducted, which showed that the airway of the patient was intact. Endoscopy of the upper gastrointestinal tract showed a bulge at 22 cm to the incisor, with a black hemispheric lustrous foreign matter at the center, which was surrounded by esophageal mucosa (Fig. 2). Endoscopy confirmed that the patient was with postoperative esophageal perforation caused by screw. CT scan showed that the right screw perforated the anterior cortex of the vertebral body and the esophagus at the T4 level (Fig. 3). However, fortunately, no mediastinal infection was observed in the CT scan.

After preoperative preparation, emergent posterior wound incision and rinsing was conducted. The T4 screw was removed, Vacuum Sealing Drainage (VSD) was performed, and jejunum catheterization was used for enteral nutrition. The patient was transferred to the intensive care unit (ICU) after the operation. The vital signs of the patient were stable on the day after operation. After consulting with the physicians or surgeons from the departments of Gastroenterology, ICU, Thoracic Surgery, and Spinal Surgery, the patient was transferred back to our department 2 days later, while symptomatic and supportive treatment, including enteral nutrition, anti-infection, infusion of human serum albumin, VSD, and continuously enhanced anti-tuberculosis were conducted. Endoscopy at 1 month later showed that the perforation of esophagus was completely healed, while the lumen dimeter of the esophagus was about 50% lower than that of normal esophagus. However, fortunately, the patient had no symptoms of dysphagia or pain when swallowing, and thus no esophageal dilation was performed. The patient’s wound was with infection when the esophageal perforation was discovered. Bacterial culture showed that the infection was caused by *Escherichia coli*. After continuous treatment with sensitive antibiotics for 2.5 months and 5 times of VSD vacuum aspiration, the infected wound recovered gradually.

**Fig. 4:**
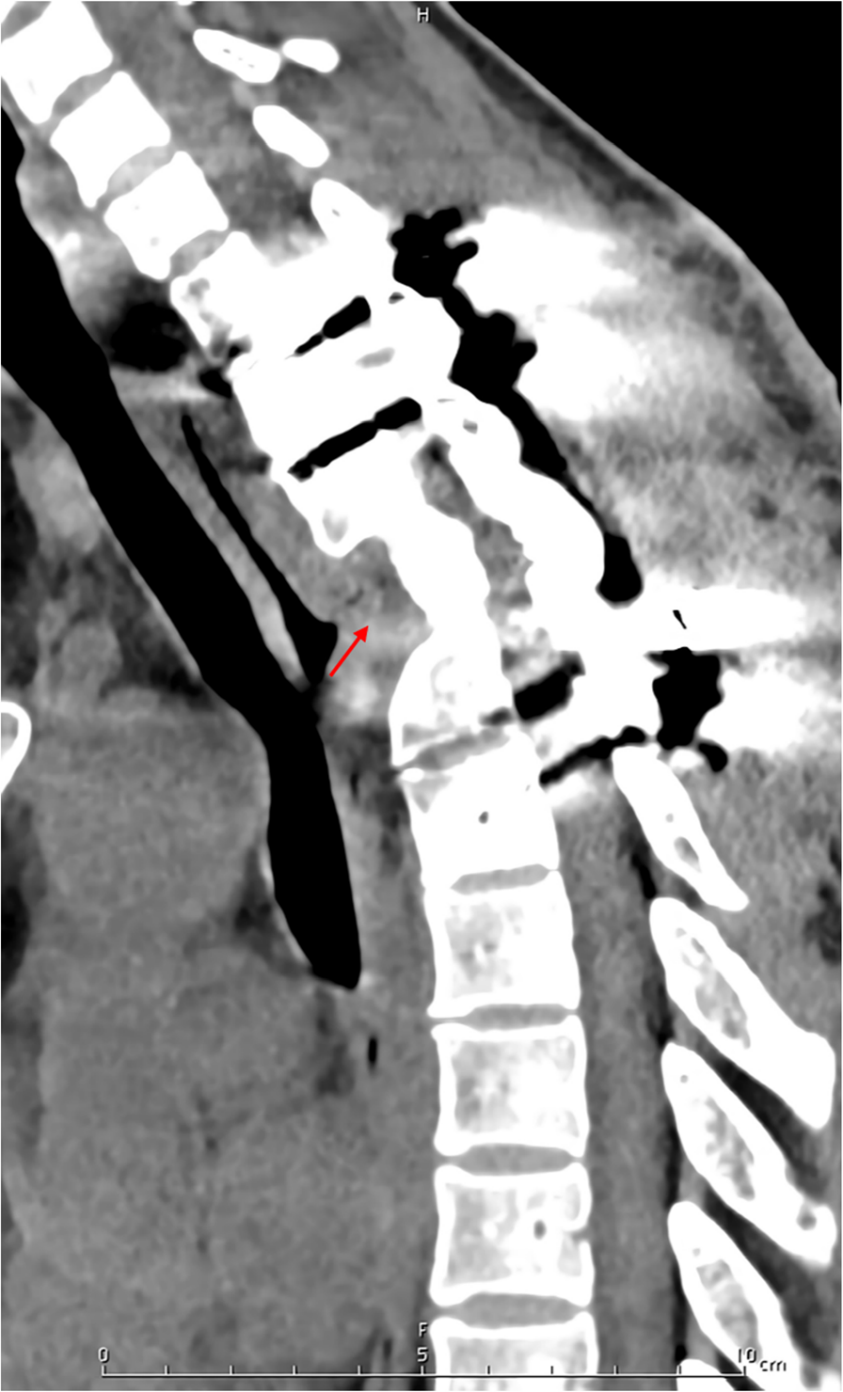
18 months after operation, CT examination showed that T2–4 had complete osseous fusion without dead bone existed

After treated with the enhanced tetrad anti-tuberculosis strategy for 3 months, the patient was further treated with isoniazid, rifampicin, and pyrazinamide for 15 months for anti-tuberculosis. In addition, imaging and blood examinations were also conducted for the patient every 3 months. The re-examination at 18 months after operation showed tuberculosis clinical manifestations of the patient disappeared, 3 continuous ESR and CRP examinations showed normal results, CT scan showed the union of bone graft, and the upper thoracic spinal abscess disappeared, while no sequestrum was found (Fig. 4). These findings demonstrated that the upper thoracic spinal tuberculosis met the criteria of clinical healing.

## Discussion and conclusion

Screws were too long should be the primary cause of severe esophageal perforation in this case. Previously, Sariyilmaz et al. [5] and Marouby et al. [13] independently reported one scoliosis case with delayed esophageal perforation following the screw placement in T4 segment (one at 3 years after operation, while the other at 10 years after operation). The esophageal perforations in both cases were caused by the long-term friction of esophagus due to the too-long screw, which perforated the anterior cortex of vertebrae and contacted with the esophagus. In another case reported by O’Brien et al. [14], the esophagus was damaged due to the oppression of the too-long screw which was placed in the T3 segment. Fortunately, the damage was detected early and endoscopy showed no sign of esophageal perforation. It was apparent that the esophageal perforation and damages following posterior pedicle screw placement in upper thoracic spine in all reported cases were caused by the too long screws.

Kwan et al. [15] used CT scan to examine 2020 pedicle screws, and found that the rate of perforating anterior cortex of vertebral body was 6.4%. Belmont et al. [16] assessed the placements of 279 pedicle screws in thoracic spine, of which the postoperative CT scan showed that the mean length of perforating anterior cortex of vertebral body was 1.7 mm (1–4 mm) in 6% screws. The highest rate of perforating anterior cortex of vertebrae was in the T1-T4 vertebral bodies, which was in agreement with the findings of this case report that the T4 pedicle screw perforated the esophagus. In contrast to the upper thoracic spine, the too-long screw could also potentially perforate the right azygos vein, parietal pleura, left thoracic aorta, and esophagus [13]. Sariyilmaz et al. [5] suggested that screws with the length of > 35 mm should not be used in upper thoracic spine. Di Silvestre et al. [11] were even more careful, as they suggested that the length of the screws should not be longer than 25–30 mm in upper thoracic spine. In this case, the length of the screw that perforated the esophagus was 35 mm. We suggest that for treating upper thoracic spinal tuberculosis involving the destruction of vertebral bodies and surrounding soft tissues, screws with the length of ≤30 mm should be selected as possible.

Using preoperative CT scan to predict the length from the insertion site to the anterior cortex of vertebral body could possibly prevent such complication. Still, preoperative planning could not completely eliminate the risk of screw complications, as the screw placement during the operation may not be as scheduled due to the deviation of the site and angle of the insertion. It has been demonstrated that intraoperative imaging could reduce the pedicle screw-related complications [12]. However, it is difficult to display the anterior margin of the upper thoracic spinal vertebral body by X-ray imaging in prone position [14]. A meta-analysis investigated the safety profiles in 7533 pedicle screws, which showed that the accuracy rate of intraoperative CT and 2-dimensional X-ray were 90 and 85%, respectively. The difference is even higher in the upper thoracic spine [17]. Therefore, we suggest that for pedicle screw placement for upper thoracic spinal tuberculosis, preoperative CT scan should be conducted to accurately measure the anatomical parameters, and intraoperative CT navigation should also be adopted to reduce the risk of complications caused by the screws.

In recent years, robot techniques have been introduced to the field of spinal surgery [18]. The accuracy rate of robot-assisted pedicle screw placement was over 98%, which could evidently reduce the incidence of complications [19–21]. Therefore, robot-assisting techniques could also be used to reduce the risk of screw-related complications.

Upper thoracic spinal tuberculosis is a chronic inflammatory disease, in which the characteristics of the tuberculous lesions could also increase the risk of esophageal damages caused by pedicle screw placement. Cardoso et al. [22] conducted T1-T4 CT scan for 20 patients, and found that esophagus was with the highest risk of nosocomial damages regardless of the vertebral body level. The mean distance from the tip of left screws to esophagus was lower than that of the right screws, while the T2 vertebrae had the highest risk. In the present case, however, the right T4 screw perforated the anterior cortex of vertebrae and caused esophageal perforation. It could not only be associated with the fact that the right screw at T4 level was too long, but also that the tuberculous lesions invaded the esophagus and reduced the elasticity and increased the fragility of the tissues, which made the esophagus tend to be damaged easier. In addition, the lesions also changed the anatomical relationship between esophagus and surrounding tissues, and the paravertebral abscess pushed the esophagus to the right side, which further increased the risk of esophageal perforation by pedicle screw.

This case report first reported the esophageal perforation following posterior pedicle screw placement for upper thoracic spinal tuberculosis. This complication is extremely rare, which could directly affect the lives of patients, and the mortality rate is as high as 20% [23]. Both the two cases reported in previous studies were delayed perforation, and the posterior wound had already completely healed, thus the treatment was relatively easier. The case reported by Marouby et al. [13] achieved a good outcome even without removing the screw. While in this case, the spinal tuberculosis patient was accompanied with esophageal perforation and wound infection, and thus the treatment was relatively tricky. In addition to enhanced tetrad anti-tuberculosis treatment, we also treated the wound infection and esophageal perforation separately. The wound improved after surgical cleaning, rinsing, drainage, and VSD vacuum aspiration. In principle, because of the limited injury of esophageal wall in esophageal perforation caused by screws alone, if there were no systemic infection symptoms after removing the screw, appropriate treatment and nutritional support including careful observation, forbidding oral food intake, intravenous injection of broad-spectrum antibiotics, and application of proton-pump inhibitor should be sufficient for the treatment [24]. In this case, enteral nutrition by jejunum catheterization was conducted for the patient for 1 month, and the esophageal perforation healed completely, which demonstrated the validity of the principle. The prognosis of esophageal perforation is mainly dependent on the presence of mediastinal infection and its treatment effectiveness [25]. Once combined with mediastinal infection, the mortality of patient increases dramatically. Fortunately, the esophageal perforation detected in our patient was in time (On the Day 7 after operation), and no mediastinal infection occurred. The delayed diagnosis of perforation could lead to irreversible outcomes and even endanger the life of patient.

In summary, for patients with upper thoracic spinal tuberculosis, the direct invasion of esophageal wall by tuberculous lesions and chronic oppression of esophagus by paravertebral abscess could lead to ischemic necrosis and thinning of esophageal wall, and the destruction of anterior cortex of vertebral body, all of which increase the risk of esophageal perforation. It is necessary to carefully evaluate the preoperative imaging data and clarify the risky anatomic structures. In addition, the distance from the screw insertion site to anterior cortex of vertebral body should also be measured, and screws with the length no more than 30 mm should be selected. Navigation or robot-assisted techniques could also be adopted if possible, which could prevent the esophageal damages caused by the inserted screws. For patients with the symptoms and signs highly suggesting esophageal perforation, early diagnosis and early treatment should be conducted as early as possible to prevent devastating outcomes such as mediastinal infection.

## Data Availability

All relevant data was presented within the manuscript and the datasets used and/or analyzed during the current study are available from the corresponding author on reasonable request.

## Abbreviations

CT: Computed tomography
VSD: Vacuum sealing drainage
T: Thoracic vertebra
T-STOP.TB: T cell enzyme-linked immunospot assay for tuberculosis
NSAIDs: Nonsteroidal anti-inflammatory drugs
MRI: Magnetic resonance imaging
ESR: Erythrocyte sedimentation rate
CRP: C-reactive protein
ASIA: American spinal injury association
ICU: Intensive care unit
